# Dairy Heifers Naturally Exposed to Fasciola hepatica Develop a Type 2 Immune Response and Concomitant Suppression of Leukocyte Proliferation

**DOI:** 10.1128/IAI.00607-17

**Published:** 2017-12-19

**Authors:** John Graham-Brown, Catherine Hartley, Helen Clough, Aras Kadioglu, Matthew Baylis, Diana J. L. Williams

**Affiliations:** a Department of Infection Biology, Institute of Infection and Global Health, University of Liverpool, Liverpool, United Kingdom; b Department of Epidemiology and Population Health, Institute of Infection and Global Health, University of Liverpool, Liverpool, United Kingdom; c Department of Clinical Infection, Microbiology and Immunology, Institute of Infection and Global Health, University of Liverpool, Liverpool, United Kingdom; d National Institute of Health Research, Health Protection Research Unit in Emerging and Zoonotic Infections, University of Liverpool, Liverpool, United Kingdom; Washington State University

**Keywords:** multivariable regression modeling, natural challenge, parasitology, veterinary immunology, veterinary vaccine development, zoonotic infections

## Abstract

Fasciola hepatica is a parasitic trematode of global importance in livestock. Control strategies reliant on anthelmintics are unsustainable due to the emergence of drug resistance. Vaccines are under development, but efficacies are variable. Evidence from experimental infection suggests that vaccine efficacy may be affected by parasite-induced immunomodulation. Little is known about the immune response to F. hepatica following natural exposure. Hence, we analyzed the immune responses over time in calves naturally exposed to F. hepatica infection. Cohorts of replacement dairy heifer calves (*n* = 42) with no prior exposure to F. hepatica, on three commercial dairy farms, were sampled over the course of a grazing season. Exposure was determined through an F. hepatica-specific serum antibody enzyme-linked immunosorbent assay (ELISA) and fluke egg counts. Concurrent changes in peripheral blood leukocyte subpopulations, lymphocyte proliferation, and cytokine responses were measured. Relationships between fluke infection and immune responses were analyzed by using multivariable linear mixed-effect models. All calves from one farm showed evidence of exposure, while cohorts from the remaining two farms remained negative over the grazing season. A type 2 immune response was associated with exposure, with increased interleukin-4 (IL-4) production, IL-5 transcription, and eosinophilia. Suppression of parasite-specific peripheral blood mononuclear cell (PBMC) proliferation was evident, while decreased mitogen-stimulated gamma interferon (IFN-γ) production suggested immunomodulation, which was not restricted to parasite-specific responses. Our findings show that the global immune response is modulated toward a nonproliferative type 2 state following natural challenge with F. hepatica. This has implications in terms of the timing of the administration of vaccination programs and for host susceptibility to coinfecting pathogens.

## INTRODUCTION

The liver fluke (Fasciola hepatica) is a parasitic trematode of global importance that is capable of infecting a wide range of vertebrate hosts, including humans. Fasciolosis is considered a major issue for global food security, with over 600 million sheep and cattle thought to be infected worldwide (1). In cattle, the clinical presentation ranges from severe, acute disease to chronic disease depending on the intensity and stage of infection, although both types of disease result in significant morbidity and/or production losses. Subclinical infections reduce weight gain, fertility, and milk yield, which impacts the economic viability of farm production systems (2, 3) and may last for months or years if untreated (4).

Both the incidence and prevalence of infection have increased across Europe over the last decade. This is largely attributed to climatic changes, namely, increases in ambient temperatures and rainfall, favoring the development of both F. hepatica and its intermediate snail host, Galba truncatula. As a result, the range and prevalence of infection are increasing spatially and temporally, with changes in climate being projected to further increase the prevalence across Europe in the coming decades (5).

The control of fasciolosis in livestock is currently based on a limited number of anthelmintics, of which triclabendazole (TCBZ) has been the most heavily used due to its efficacy against both adult and migratory juvenile stages of the parasite (6). TCBZ-resistant F. hepatica infections in livestock are now widely reported (7–12). There is therefore a need to develop novel approaches to control fasciolosis, with vaccination being proposed as a potential adjunctive measure.

Current vaccine trials are focused on several immunodominant parasite antigens, including cathepsin L proteases, glutathione *S*-transferase (GST), fatty acid binding proteins (FABPs), and leucine aminopeptidase (LAP), which have been tried in a number of formulations and host species (cattle, sheep, and goats) in both native and recombinant forms, with levels of protection ranging from 0 to 72% (13, 14). In cattle, as with other ruminant species, protection is often only partial, with reductions in fluke burden, egg output, and viability being observed in vaccinated animals compared to unvaccinated controls (15, 16). In these circumstances, mathematical modeling has shown that such partial protection must be induced in at least 90% of the herd to have a meaningful impact on disease control (17).

Where vaccine-induced protection is demonstrated, it has been shown to correlate with parasite-specific IgG2 isotype antibody titers and avidity and a reduction in arginase activity in CD14^+^ blood monocyte-derived macrophages, suggesting that the presence of a cell-mediated type 1 response is an important component of vaccine-induced protection against F. hepatica (15, 18). Such responses, however, are not typically associated with infection in unvaccinated animals.

Epidemiological evidence shows that the prevalence of F. hepatica infection increases with age (19), while chronically infected cattle remain susceptible to superimposed experimental infections (20), suggesting an absence of any protective immunity. Studies investigating the immune responses of cattle experimentally infected with F. hepatica initially showed a proinflammatory response progressing over the course of infection toward a polarized, nonproliferative state. Parasite-specific interleukin-2 (IL-2), IL-4, and gamma interferon (IFN-γ) production is observed in hepatic lymph nodes at 10 to 14 days postinfection (21), and parasite-specific IL-2 and IFN-γ production is also detected in peripheral blood mononuclear cells (PBMCs) from 1 to 3 weeks postinfection (wpi), becoming absent by 5 wpi (22, 23). Similarly, mitogen- and parasite-specific stimulated PBMC proliferation peaks at around 2 wpi before returning to preinfection levels (22–25). Thereafter, responses progress toward a nonproliferative state associated with type 2 cytokines, an IgG1 isotype antibody response, and eosinophilia (20, 24, 26). By 10 to 12 wpi, when the parasite has reached the bile ducts and matured, there is a lack of mitogen- or parasite-specific lymphocyte proliferation and an upregulation of IL-10 and transforming growth factor β (TGF-β) (27, 28). It has been suggested that this fluke-induced modulation of the immune response facilitates its long-term survival within the host (29) and may also impact the host's susceptibility to other, coinfecting pathogens such as Salmonella enterica serovar Dublin (30) and Mycobacterium bovis, the causative agent of bovine tuberculosis (31, 32).

Current vaccine development programs are based on the assumption that immune responses observed in experimentally infected cattle are representative of those in naturally infected cattle. However, little is known about the immune responses associated with naturally acquired F. hepatica infections, specifically the early stages of infection, and the extent to which parasite-induced immunomodulation is induced. This may have implications for how vaccines are delivered in the field and for understanding how this parasite may affect host susceptibility to coinfecting pathogens. The aim of this study was therefore to define the immune responses in cohorts of dairy heifers on United Kingdom farms under typical management conditions and naturally exposed to challenge with F. hepatica.

## RESULTS

### Fecal egg counts and antibody responses.

All calves remained healthy throughout the study, with no clinical signs as a result of F. hepatica infection, nematode burden, or other disease being observed. On each farm, the adult milking herd was monitored monthly by using a sample of milk from the bulk tank. All three herds tested positive on every occasion, with bulk milk tank (BMT) antibody values ranging from 33 to 50% positivity, 54 to 61% positivity, and 79 to 114% positivity for farms A, B, and C, respectively.

**FIG 1:**
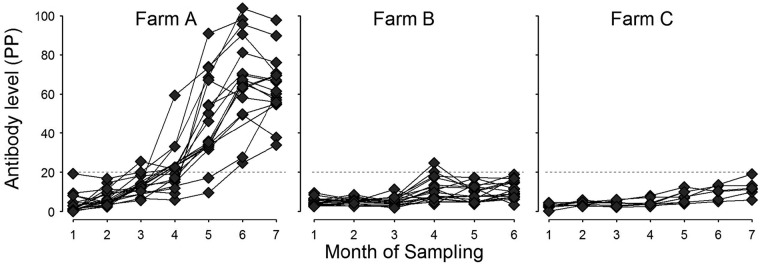
Antibody PP values of individual animals over the study period for the three dairy farms (farms A to C). The diagnostic positive cutoff value (PP = 20%) is denoted by a horizontal line. Month of sampling denotes the time point for each sequential visit to each farm.

Seroconversion was observed in all 17 calves on farm A; percent positivity (PP) values increased over the course of the study, reaching 37 to 98% at the final time point (Fig. 1). Fluke eggs were detected in 10 of the 17 animals from farm A by the final time point, with counts in all cases of <1 egg per g (epg) of feces. Paramphistome eggs were also observed in low numbers in seven calves at the final time point. Six of these calves were positive for both F. hepatica and paramphistome eggs.

PP values for the cohorts from farms B and C remained negative throughout the study, except for two animals from farm B that had positive PP values at a single time point. These PP values were close to the cutoff (20.3 and 24.7%), and samples taken from these two calves on every other occasion were negative, suggesting that these results were false-positive results. All calves from farms B and C remained negative for fluke eggs for the duration of the study.

Low numbers of nematode eggs were detected in calves on all three farms. Only two positive samples, with counts of 50 epg, were detected on farm A at a single time point. Both trichostrongyle and Nematodirus species eggs were observed intermittently in 15/17 calves from farm B from August onwards, and trichostrongyle eggs were observed intermittently in 4/8 calves from farm C from July onwards.

### Immunological correlates of infection.

**FIG 2:**
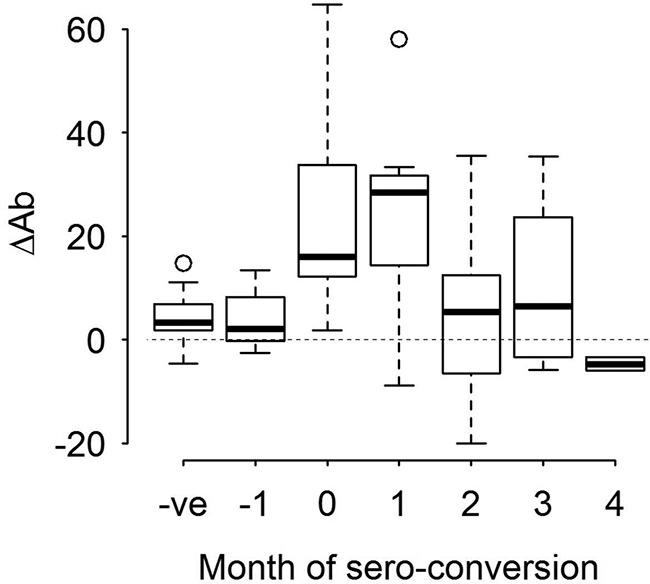
Change in antibody PP values (ΔAb) over the course of infection for farm A. Positive values indicate an increase in the Ab PP value from one time point to the next, and negative values denote a decrease in the Ab PP value compared to that for the previous month. Values from 1 month prior to seroconversion (−1) are considered separately from other seronegative values (−ve), as infection may have been present at this time point, but the animals had not seroconverted. In experimentally infected animals, seroconversion occurs at 2 to 4 weeks postinfection (42).

Twenty-three immune and four signalment parameters were measured monthly for each calf, yielding over 1,100 separate data points. To analyze these data, we used multivariable linear mixed-effect modeling. Two response variables were used: (i) the antibody PP value (Ab) was used to indicate infection progression, and (ii) the change in the antibody value (ΔAb) after seroconversion was used to indicate the point of first exposure and the early stage of infection. ΔAb was used because the increases in PP values were highest at the point of seroconversion (Fig. 2).

### (i) Association between Ab and immune responses.

The outputs from the three models using Ab as the response variable (lme_1, lme_2, and lme_3) are shown in Table 1. Statistically significant positive associations between Ab (response variable) and days of exposure were found in all three models, showing that the longer the calves were grazing, the greater the likelihood of infection. A significant negative relationship between Ab and somatic antigen (SomAg)-specific PBMC proliferation was also observed in all three models.

**TABLE 1:** Multivariable linear mixed-effect model outputs for anti-F. hepatica antibody PP values as response variables^*a*^

Model, type of data used	Explanatory variable (*x*)	Coefficient value (β)	SE	*P* value
lme_1, all data	Farm B	−0.687	0.077	<0.001
	Farm C	−0.874	0.087	<0.001
	Days of exposure	0.005	<0.001	<0.001
	Eϕ/ml	0.0001	0.00004	0.024
	CD8 cells/ml	0.0002	<0.0001	0.016
	WC1 cells/ml	−0.0001	0.00004	0.028
	SomAg	−0.006	<0.003	0.024
lme_2, farm A (positive cohort)	Days of exposure	0.027	0.003	<0.001
	SomAg	−0.012	0.005	0.039
	[ConA] IFNγ	−0.0001	<0.00006	0.026
	[ConA] IL-4	0.0005	0.0002	0.039
	[SomAg] IL-5	0.003	0.001	0.012
lme_3, farms B and C (negative cohorts)	Farm C	−0.201	0.086	0.029
	Days of exposure	0.004	<0.001	<0.001
	CD4 cells/ml	−0.0001	0.00004	0.010
	CD8 cells/ml	0.0004	0.0001	<0.001
	SomAg	−0.007	0.003	0.042

a Statistically significant (*P* < 0.05) explanatory variables (*x*) are shown with corresponding coefficient (β) and standard error (SE) values to describe their respective relationships with Ab. Values are rounded to 3 decimal places, or 1 significant figure. For cytokine data, brackets indicate which antigen-stimulated culture condition is referenced, i.e., ConA or SomAg.

When data from all three farms were used (lme_1), statistically significant negative associations were found for farms B and C relative to farm A. Statistically significant positive associations were detected for eosinophil and CD8^+^ PBMC counts, and a negative coefficient was estimated for peripheral blood WC1^+^ cell counts.

When the immune response data from farm A were used (lme_2), there was a negative association between Ab and concanavalin A (ConA)-induced IFN-γ production and a positive association with ConA-induced IL-4 and SomAg-induced IL-5 transcription.

The model with data from fluke-negative cohorts on farms B and C (lme_3) showed a significant increase in peripheral CD8^+^ T-cell counts and a corresponding decrease in CD4^+^ T-cell counts.

### (ii) Association between ΔAb and immune responses.

When the change in the PP value (ΔAb) was used to indicate the time of exposure (lme_4) (Table 2), there was a significant negative association between ΔAb and days of exposure, suggesting that infection occurred early after turnout and that ΔAb is a good measure of early infection. A negative association was also observed for SomAg-specific PBMC proliferation, while positive associations were observed for tegument antigen (TegAg)-specific PBMC proliferation, ConA-stimulated IL-4 production, and SomAg-stimulated IL-5 transcription.

**TABLE 2:** Multivariable linear mixed-effect model outputs for the change in antibody PP values as a response variable^*a*^

Model, type data used	Explanatory variable (*x*)	Coefficient value (β)	SE	*P* value
lme_4, farm A (postseroconversion)	Days of exposure	−0.354	0.107	0.004
	SomAg	−2.794	1.211	0.034
	TegAg	2.254	1.064	0.049
	[ConA] IL-4	0.012	0.050	0.033
	[SomAg] IL-5	0.071	0.029	0.026
lme_5, farms A to C (seronegative values)	Farm B	−12.009	4.675	0.015
	Farm C	−14.760	5.096	0.006
	CD8 cells/ml	0.027	0.009	0.008
	SomAg	−0.621	0.276	0.027
	TegAg	0.712	0.248	0.005

a Statistically significant (*P* < 0.05) explanatory variables (*x*) are shown with the coefficient (β) and standard error (SE) values to describe their respective relationships with ΔAb. Values are rounded to 3 decimal places. For cytokine data, brackets indicate which antigen-stimulated culture condition is being referenced, i.e., ConA or SomAg.

When seronegative data from all farms were used (lme_5) (Table 2), there was a significant negative association with SomAg-specific PBMC proliferation and a positive association with peripheral blood CD8^+^ counts.

### Cellular proliferation and cytokine responses.

**FIG 3:**
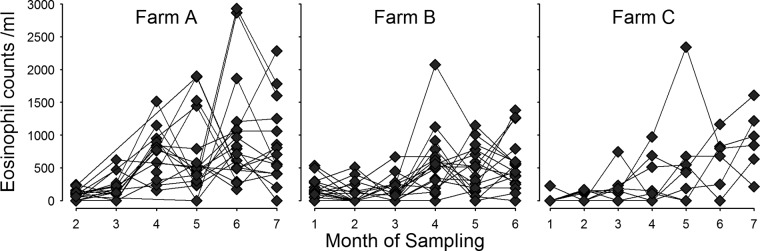
Peripheral blood eosinophil counts for individual animals over the study period for the three dairy farms (farms A to C). Month of sampling denotes the time point for each sequential sampling visit.

Eosinophil counts increased in all the animals on all three farms over the course of the study (Fig. 3). PBMC proliferation responses were variable both between animals and for individual animals at different time points. The highest parasite-specific proliferation responses were observed for animals from farm A.

A similar variation was observed for the cytokine responses of animals from farm A. An increase in ConA-stimulated IFN-γ production was observed following seroconversion, and there was also evidence of ConA-stimulated IL-4 production. No IFN-γ production was detected in response to stimulation with SomAg, while only low levels of IL-4 production were observed. IL-2 and IL-5 transcription levels were similarly variable between and within individuals over the course of the study in response to stimulation with both ConA and SomAg.

No significant difference was found between the levels of TGF-β expression in early- and late-stage infections for either medium control or SomAg-stimulated PBMC cultures (*P* = 0.791 and 0.828, respectively), nor was any significant difference found between TGF-β levels in medium control and SomAg-stimulated cultures at either time point (*P* = 0.291 and 0.306, respectively).

## DISCUSSION

In this study, we demonstrate that dairy calves develop a polarized, nonproliferative type 2 response following primary natural challenge with F. hepatica. Previous studies using experimentally infected calves described an initial inflammatory response comprised of mixed-cytokine (IFN-γ and IL-4) production and antigen-specific proliferation in PBMC cultures, which subsides at 4 to 6 weeks postinfection. Thereafter, parasite-specific IgG1 and IL-4 responses are detected, indicating polarization toward a type 2 immune response as infection progresses (20, 22, 23, 33). Our analysis of naturally infected calves shows an association with increased eosinophil counts, IL-4 production, and IL-5 transcription and decreased IFN-γ production, indicating polarization toward a type 2 response as infection progresses. Furthermore, the increased IL-4 and decreased IFN-γ production by ConA-stimulated PBMCs suggest that polarization of the global T-cell response is present. This is consistent with previous findings in experimentally infected cattle (32, 34). The presence of a generalized type 2 immune environment may help explain why calves are less able to respond effectively to coinfecting pathogens. For example, cattle infected with F. hepatica are more susceptible to Salmonella Dublin infection (30, 32).

Our findings differ from those obtained from experimentally infected calves, however, in the rate and stage at which immune modulation is observed. Experimentally infected cattle have increased IL-2 and IFN-γ production in the first 2 to 3 weeks postinfection (22, 23), while our study suggests that the early stages of natural infection are associated with increased IL-4 production and IL-5 transcription. Similarly, D. G. Clery and G. Mulcahy (22) showed parasite-specific proliferation within 2 to 3 weeks postinfection in experimentally infected calves, while our study detected a significant negative association between SomAg-specific PBMC proliferation at all stages of infection. Early proliferative responses were observed in only 3/17 animals. For this study, samples were taken monthly; hence, some transient early proinflammatory responses may have been missed. Nonetheless, our findings suggests that, unlike experimental infections, polarization of the immune response in naturally acquired infection is present very early during infection.

These differences may relate to the infectious challenge administered in experimental infections compared to those encountered under field conditions. Most experimental infections have used either a single-dose or a “trickle”-type infection, where boluses of several hundred to up to 1,000 metacercariae are administered per dose (26, 35). The epidemiology of F. hepatica in temperate regions typically results in small numbers of metacercariae being present on pastures early in the spring, followed by increasing numbers of metacercariae appearing on pastures toward the end of the grazing season (36). Hence, the administration of a large infectious challenge to immunologically naive cattle may not be representative of normal field conditions and may result in a more profound innate cellular response during the early stages of experimental infection.

Our results suggest that challenge at the start of the grazing season was not associated with detectable proinflammatory or proliferative responses. The immune modulation induced during this primary challenge may also prevent any subsequent proinflammatory, proliferative responses developing later in the season when animals are exposed to a more substantial infectious challenge as pasture contamination increases. These findings have implications for vaccine programs, since current efforts are focused on enhancing cell-mediated type 1 responses (15, 18). Our results suggest that the efficacy of such vaccines would be negatively impacted if they were administered following natural exposure to F. hepatica under field conditions. These vaccines would therefore need to be administered and be fully effective prior to any exposure to infection.

In experimentally infected calves, a systemic regulatory response develops during the chronic stages of infection, characterized by increased parasite-specific IL-10 and TGF-β production by PBMCs (27). In this study, we found no significant association between infection status and parasite-specific IL-10 or TGF-β production by PBMCs. Again, this may relate to differences in the ways in which the calves were exposed to infection, although it is possible that such regulatory responses were present in local inflammation and regional lymph nodes, as this was observed previously for cattle and sheep harboring naturally acquired, chronic F. hepatica infections (28, 37).

Eosinophilia is a feature of many helminth infections. Calves on farm A were treated with three doses of ivermectin over the course of the grazing season, and nematode egg counts remained at or close to zero throughout the study period. The eosinophilia observed in this cohort was most likely a response to F. hepatica rather than nematode infection, particularly since there was a significant positive relationship between fluke Ab PP values and both peripheral eosinophil counts and SomAg-stimulated IL-5 transcription. This is consistent with previous findings of eosinophilia in F. hepatica-infected cattle (26, 38).

Both fluke infection and specific fluke antigens are known to have immunosuppressive and/or modulatory effects on both the innate and adaptive immune responses (20, 27, 33, 39). Differences between the relationships of SomAg and TegAg PBMC proliferation and the early stages of F. hepatica exposure (ΔAb) may be the result of differences in the compositions of these two antigen fractions and their functions *in vivo*. Overall, however, our results show that in naturally infected calves, there is an absence and/or suppression of parasite-specific T-cell proliferation.

The negative relationship between Ab and WC1^+^ PBMC counts is most likely to be due to age and the maturation of the immune system. Numbers of WC1^+^ γδT cells are known to be decreased in the peripheral blood as cattle mature. WC1^+^ cell populations constitute approximately 15% of PBMCs in calves aged 3 to 12 months, decreasing to around 5% by 3 years of age (40). In our study, animals ranged in age from 90 to 377 days at the beginning of the study and were 310 to 587 days of age by the end of the study. The respective mean WC1^+^ PBMC counts were 20% (6 to 32%) and 6% (0.5 to 24%). Similarly, the positive relationship between Ab and CD8^+^ PBMC counts may also signify the normal maturation of the immune system rather than a direct impact of infection, since this was also observed in uninfected animals.

In spite of evidence from the adult milking herd showing that F. hepatica infection was present on all three farms, the calves on farms B and C showed no evidence of infection. In both cases, these calves were kept in different parts of the farm and did not share pastures with the adult cows. In contrast, the cohort on farm A was managed in a rotational grazing system that included pastures grazed by adult cattle. This suggests that the fields used for calves on farms B and C had no habitat suitable for the intermediate host, Galba truncatula, and were not contaminated with metacercariae.

In this study, multivariable linear mixed-effect regression models were used to analyze a longitudinal set of data from cattle naturally exposed to F. hepatica. This analysis was useful since it allowed the simultaneous assessment of multiple immunological variables to identify the key parameters associated with infection. To the best of our knowledge, this is the first time that such techniques have been used to analyze complex immunological responses to natural infection in livestock and may represent a potentially useful template for future studies.

In conclusion, by analysis of a longitudinal data set using multivariable linear mixed-effect regression analysis, we have demonstrated that natural exposure to F. hepatica in dairy heifers results in a generalized type 2 immune state with the concomitant suppression of proliferation responses. This has significance for both vaccine administration programs and susceptibility to other common coinfecting pathogens of livestock.

## MATERIALS AND METHODS

Three commercial dairy farms were recruited for this study. These farms were identified as being F. hepatica positive through BMT antibody enzyme-linked immunosorbent assay (ELISA) results (41) and positive composite fecal egg counts in adult cattle. On each farm, cohorts of replacement dairy heifer calves were recruited (*n* = 42; 17, 17, and 8 animals from farms A, B, and C, respectively) and were 90 to 377 days of age (mean, 218.5 days; standard deviation [SD], ±62.0 days). These animals had been housed since birth, so they had not been exposed to F. hepatica. This was confirmed prior to turnout by fecal fluke egg counts and an anti-F. hepatica IgG serum antibody ELISA (42). Negative ELISA results indicated that no maternally derived antibodies were detectable at turnout.

Animals were sampled monthly over the course of their first grazing season from turnout in spring (April to May) through to housing in autumn (October to November) of 2013. On each occasion, blood was collected via jugular venipuncture into plain and EDTA-coated vacutainers, and fecal samples were collected rectally. BMT samples were also taken at each visit to assess changes in levels of exposure within the milking herd.

All the procedures used in this study were approved by the University of Liverpool Veterinary Research Ethics Committee (VREC100) and adhered to the conditions of the project license granted by the United Kingdom Home Office (HOL PPL40/3621). All farm data were stored in accordance with the United Kingdom Data Protection Act (1998).

### F. hepatica serum and BMT IgG antibody ELISA.

Tubes containing clotted blood were centrifuged at 2,000 × *g* for 5 min, and serum was collected, stored at 4°C, and tested for fluke-specific antibody within 5 days. A positive cutoff of 20% positivity was used, giving a diagnostic sensitivity of 95% and a specificity of 99% (42). BMT samples were analyzed by using a PP cutoff value of ≥27%, giving a diagnostic sensitivity of 96% and a specificity of 80%, as described previously (41).

### Fluke egg counts.

Fecal samples were stored at 4°C prior to analysis and examined for evidence of F. hepatica eggs according a standard sedimentation technique using 10 g of feces (43). This was performed on individual samples from the point of seroconversion onwards, with counts being done for every animal at the final time point irrespective of whether they had seroconverted. Nematode infections for all animals at each time point were assessed by fecal egg counts using the McMaster method, with a sensitivity of 50 epg (44).

### Preparation of F. hepatica antigens.

Adult F. hepatica tegument and somatic antigen fractions for use in *in vitro* PBMC stimulation assays were prepared by using previously described methods (23, 45); live adult flukes were collected from infected livers, incubated overnight to purge cecal contents, and then washed three times in Dulbecco’s phosphate-buffered saline (D-PBS) (Sigma-Aldrich, St. Louis, MO, USA).

TegAg was prepared by placing flukes in D-PBS with 1% Nonidet P-40 (BDH Chemicals, Poole, UK), at 1 ml per fluke, and agitating the mixture for 1 h at 4°C. Pierce Detergent Removal spin columns (Thermo Fisher Scientific, Waltham, MA, USA) were used to remove the Nonidet P-40 detergent.

SomAg was prepared from tegument-depleted flukes. These flukes were washed in ice-cold D-PBS and snap-frozen overnight at −80°C. Flukes were then homogenized and diluted in D-PBS, at 0.5 ml per fluke, and left to settle overnight at 4°C. The supernatant was collected and centrifuged at 12,000 × *g* for 30 min at 4°C.

Both antigens were filter sterilized and shown to contain negligible levels of endotoxin at tissue culture concentrations (Thermo Fisher Scientific) (46). Protein concentrations were estimated by using a Bradford assay (Thermo Fisher Scientific), and aliquots were stored at −80°C.

### Hematology, PBMC purification, and flow cytometry.

Total and differential leukocyte counts were performed on EDTA-treated whole blood using a hemocytometer and thin blood smears, respectively, and used to calculate absolute counts for each leukocyte phenotype per milliliter of blood.

PBMCs were isolated from whole blood in a lateral flow hood using Optiprep (Sigma-Aldrich, St. Louis, MO, USA) according to the manufacturer's recommendations. Optiprep was added to, and mixed with, EDTA-treated whole blood, at 1.3 ml of Optiprep per 10 ml of blood, in a 50-ml Falcon tube, onto which 1 ml 20 mM Tricine-buffered saline was layered. Samples were centrifuged at 1,000 × *g* for 35 min at 20°C with the brake off, after which the middle aqueous layer containing PBMCs was harvested and washed in PBS with 0.1% EDTA (Lonza, Basel, Switzerland) to a maximum volume of 20 ml. Samples were centrifuged at 350 × *g* for 8 min at 20°C. The resulting supernatants were discarded, and cell pellets were resuspended in 2 ml of 0.9% NH_4_Cl hemolysis buffer and gently agitated for 1 min at room temperature. Samples were then washed in 20 ml PBS–EDTA and centrifuged at 150 × *g* for 8 min at 20°C twice, with purified PBMCs then being resuspended and prepared for flow cytometry, proliferation, and cytokine assays, as described previously (47).

One-color indirect immunofluorescence labeling was performed on PBMCs as previously described (47), with lineage-specific monoclonal antibodies to identify bovine CD4^+^ and CD8^+^ (48), WC1^+^ (49), and CD14^+^ (50) subsets. Subpopulations of leukocytes were analyzed by using a MACSQuant analyzer (Miltenyi Biotech Ltd.). PBMCs were isolated through gating of forward- and side-scatter channels, with adjustments being made by using a *post hoc* analysis template (MACSQuantify v.2.4.1221.1; Miltenyi Biotech Ltd.) to ensure an appropriate fit for all samples. Leukocyte subpopulations were identified and quantified through fluorescein isothiocyanate (FITC)-positive fluorescence emission and used to calculate absolute counts per milliliter of blood.

### Proliferation and cytokine measurements.

PBMCs were adjusted to a concentration of 2 × 10^6^ cells per ml in RPMI with 10% fetal calf serum (FCS) and 100 μg/ml penicillin and streptomycin and incubated *in vitro* with either ConA (5 μg/ml) or F. hepatica SomAg or TegAg (both at 25 μg/ml) or as unstimulated medium controls to assess proliferative and cytokine responses. For proliferation assays, 2 × 10^5^ cells per well were cultured in triplicate in 96-well U-bottomed plates (Corning Life Sciences, USA) for 5 days at 37°C in 5% CO_2_. On the fifth day, cultures were pulsed with 1 μCi of ^3^H-labeled tritiated thymidine (PerkinElmer, Boston, MA USA) for 5 h and then harvested onto glass filter mats and embedded in scintillation wax (PerkinElmer, Boston, MA, USA). Beta-particles were measured with a MicroBeta^2^ plate counter (PerkinElmer, Boston, MA USA). The stimulation index (SI) of mitogen/antigen-stimulated cultures was calculated as the fold increase in emission counts compared to those of medium controls. If the SI for the ConA positive control was <2, proliferation values for both mitogen- and antigen-stimulated cultures were excluded from further analysis.

For cytokine assays, PBMCs were incubated with ConA (5 μg/ml) or SomAg (25 μg/ml) or as unstimulated medium controls for 48 h at 37°C in 5% CO_2_ in flat-bottomed 24-well cell culture plates (VWR, Radnor, PA, USA). Upon completion, culture supernatants were removed and stored at −20°C, with PBMCs being stored separately at −20°C in RNAlater (Sigma-Aldrich, St. Louis, MO, USA).

### Cytokine production and transcription assays.

Cytokine production and transcription were measured in animals that seroconverted (farm A).

IFN-γ and IL-4 concentrations in supernatants were measured by using commercially available ELISAs according to the manufacturer's protocols (catalog no. MCA5638KZZ and MCA5892KZZ, respectively; AbD Serotec, Raleigh, NC, USA). IL-10 production in SomAg-stimulated and medium control cultures was measured by using a previously validated sandwich ELISA (51).

Paired samples were used to investigate differences in TGF-β production in early versus chronic infections; samples for each individual animal were selected at the closest available time point to seroconversion to represent early infection and from the last available time point to represent chronic stages of infection. Bioactive bovine TGF-β in SomAg-stimulated and medium control cultures was measured by using a commercially available ELISA kit according to the manufacturer's recommendations (Promega, Madison, WI, USA) (52).

Quantitative real-time PCR (qPCR) was used to measure IL-2 and IL-5 mRNA levels against 28S housekeeper gene transcription in cultured PBMCs by using previously reported primers (NCBI accession no. AF154866, M12791, and EU915048.1, respectively). RNA extraction was performed by using the RNeasy minikit (Qiagen, Limburg, Netherlands), and the mRNA level was quantified with RiboGreen (Invitrogen Life Technologies, Grand Island, NY, USA). Genomic DNA digestion and cDNA synthesis were then performed on 1 μg of the mRNA template for each sample by using a QuantiTect reverse transcriptase kit (Qiagen, Limburg, Netherlands). qPCR analysis was performed by using a standard protocol with SYBR green (Bioline Reagents Ltd., London, UK), as described previously (53).

Samples were assayed alongside known concentration standards diluted in a 10-fold series in 100 ng/μl yeast tRNA (Invitrogen Life Technologies, Grand Island, NY, USA) to prevent aggregation. Reactions were performed by using a DNA Engine Opticon 2 continuous fluorescence detector.

A final melting curve analysis was performed from 50°C to 95°C to confirm the specificity of the amplification products. Sample copy numbers were determined by using linear regression of standard concentrations following adjustment of threshold cycle (*C_T_*) cutoff values to the log-linear phase of amplification. Results for IL-2 and IL-5 transcription are shown as relative expression levels against the value for the 28S housekeeper gene (per million copies).

### Statistical analysis.

Data analysis was performed by using multivariable linear mixed-effects models in the “nlme” package (54) in the R statistical software environment (55). Response variables (*Y*) chosen as indicators of exposure to F. hepatica were the antibody PP value (Ab) to assess responses over the course of infection and the change in PP values (ΔAb) to examine immunological parameters associated with the early stages of infection. This approach was taken because while PP values (Ab) increased over the course of the study (Fig. 1), the greatest changes in PP values (ΔAb) were seen at or soon after seroconversion (Fig. 2).

Details of the structure of each statistical model are shown in Table 3. For all models, leukocyte counts; PBMC proliferative responses; farm location; and animal age, weight, and days of exposure (days at pasture) were modeled as fixed effects (Table 3), with individual animal identity being modeled as a random effect to account for the increased relatedness of responses measured in the same animal. Models containing data from the fluke-infected cohort (farm A) only (lme_2 and lme_4) (Table 3) were analyzed to allow the inclusion of the *ex vivo* PBMC cytokine responses measured as explanatory variables, thereby giving a more in-depth analysis of the immune responses present in these animals. Raw immunological data are summarized in the supplemental material (Fig. S1 to S6).

**TABLE 3:** Linear mixed-effects model structures^*a*^

Linear mixed-effect model	Response variable (*Y*)	Box-Cox value (λ)	Type(s) of data analyzed	Initial fixed-effect explanatory variable(s) (*x*) (AIC value)	Final explanatory variables (*x*) following stepwise AIC selection (AIC value)
lme_1	Ab	−0.2	All data	Farm + days of exposure + age + wt + leukocyte counts + CD4/CD8 ratio + PBMC proliferation (44.98)	Farm + days of exposure + Eϕ + CD4 + CD8 + WC1 + SomAg proliferation + TegAg proliferation (37.99)
lme_2	Ab	0.2	Farm A	Days of exposure + age + wt + leukocyte counts + CD4/CD8 ratio + PBMC proliferation + PBMC cytokines (166.04)	Days of exposure + Eϕ + SomAg proliferation + IFN-γ [ConA] + IFN-γ [SomAg] + IL-4 [ConA] + IL-10 [Med] + IL-10 [SomAg] + IL-2 [ConA] + IL-5 [SomAg] (145.57)
lme_3	ΔAb	Nil	Seropositive values; farm A	See lme_2 (354.80)	Days exposure + CD4 + WC1 + SomAg proliferation + TegAg proliferation +IL-4 [ConA] + IL-5 [SomAg] (329.74)
lme_4	Ab	−0.2	Farms B and C	See lme_1 (38.29)	Farm + days of exposure + SomAg proliferation + Eϕ + CD4 + CD8 + WC1 (26.06)
lme_5	ΔAb	Nil	Seronegative values; farms A to C	See lme_1 (653.12)	Farm + CD4 + CD8 + CD4/CD8 ratio + SomAg proliferation + TegAg proliferation (640.33)
lme_6	ΔAb	Nil	Farms B and C	See lme_1 (580.03)	Eϕ + CD8 (561.09)

a The response variable (*Y*) indicates the subject of each respective model. The Box-Cox value (λ) is the optimal transformation factor for the response variable to ensure linear fit to the proposed fixed-effect variables. Where “Nil” is indicated, transformation was not required. For initial fixed-effect explanatory variables, “leukocyte counts” refers collectively to leukocyte count data (eosinophils [Eϕ], neutrophils, and CD4^+^, CD8^+^, WC1^+^, and CD14^+^ PBMCs) per milliliter of peripheral blood, and “PBMC proliferation” refers to PBMC proliferation data (ConA-, SomAg-, and TegAg-stimulated cultures), while “PBMC cytokines” refers to all available PBMC cytokine production and transcription data (IFN-γ, IL-4, and IL-10 production and IL-2 and IL-5 relative gene transcription). For cytokine data present as final explanatory variables, brackets indicate which antigen-stimulated culture condition is being referenced, i.e., medium control (Med), ConA, SomAg, or TegAg. Time series plots of the immunological data included in these models are included in the supplemental material.

Three models were produced by using Ab as the response variable (lme_1, lme_2, and lme_3) (Table 3). The first analysis (lme_1) included all data for all three farms over the 7-month sampling period. Model lme_2 included data from the fluke-infected cohort (farm A) only. Model lme_3 included data from fluke-negative cohorts (farms B and C) only.

For analysis of immune responses associated with ΔAb, data collected pre- and postseroconversion were considered separately to allow the small changes in PP values associated with preseroconversion and late-stage infection to be considered separately. Consequently, two models were produced by using ΔAb as the response variable (lme_4 and lme_5) (Table 3). Model lme_4 used postseroconversion data from the fluke-positive cohort (farm A) to measure changes in immune responses associated with early exposure, while model lme_5 used seronegativity data from farms A to C.

Models containing only data from seronegative measurements (lme_4 and lme_5) served to investigate potential physiological and/or age-related changes, thus avoiding incorrectly attributing such observations to F. hepatica exposure.

Prior to the linear mixed-model analysis, the relationship between response and fixed-effect explanatory variables was assessed, using a Box-Cox transformation (56), with power λ being determined by maximum likelihood (ML) analysis used to transform the response variable to ensure an appropriate linear relationship (Table 3) (57).

For models with Ab as the response variable (*Y*), an autoregressive correlation matrix of order 1 was also used to account for the relatedness of measurements resulting from repeated sampling and, in particular, the fact that measurements taken close together in time are likely to be more similar than those taken further apart in time.

Model refinement was then carried out by using a stepwise Akaike information criterion (AIC) selection method (58), using complete case data for all models being compared with MLs using the “MASS” package. The criterion of a reduction in the AIC value of ≥2 was deemed indicative of model superiority (Table 3). The selected model was then fit to all data for which complete observations were available by using restricted maximum likelihoods (REMLs). This final model was checked for goodness of fit and normality by using residual analysis and normality plots, respectively (see Fig. S7 and S8 in the supplemental material). Missing values were considered to be “dropouts completely at random” (DCAR), since animals were moved between management groups throughout the grazing season for unrelated reasons (e.g., stocking density and pasture management, etc.) (59).

Model outputs were interpreted by inspecting the coefficient (β) and associated standard error for each explanatory variable (*x*) included in the final model. The estimated coefficients indicated whether a positive or negative relationship was present between each explanatory variable (*x*) and the (transformed) response variable (*Y*), once all other explanatory variables present in the analysis had been taken into account.

To assess TGF-β expression in early- versus late-stage infection, results were analyzed by using two-tailed paired *t* tests. Differences in measurements between the medium control and SomAg-stimulated PBMC cultures for each time point were analyzed by using unpaired two-tailed *t* tests.

## Supplementary Material

Supplemental material

## Note Added after Publication

In the originally published version, all coefficient values in Table 1 incorrectly appeared as negative values. Table 1, on page 4, now contains the correct values.
