# Supplementary material for: Dairy Heifers Naturally Exposed to Fasciola hepatica Develop a Type 2 Immune Response and Concomitant Suppression of Leukocyte Proliferation
Source: Infect Immun. 2017 Dec 19;86(1):e00607-17. doi: 10.1128/IAI.00607-17 (PMC5736823; doi:10.1128/IAI.00607-17)
Supplement: Supplemental material [file supp_86_1_e00607-17__index.html]

Supplemental material 

# Dairy Heifers Naturally Exposed to Fasciola hepatica Develop a Type 2 Immune Response and Concomitant Suppression of Leukocyte Proliferation

## Supplemental material

- Supplemental file 1 -

  Fig. S1. Differential cell counts of individual animals over the study period for each farm. Fig. S2. Differential PBMC phenotype counts of individual animals over the study period for each farm. Fig. S3. PBMC proliferative responses to mitogen/antigen stimulation *in vitro* for individual animals over the study period for each farm. Fig. S4. PBMC cytokine production responses to mitogen/antigen stimulation *in vitro* for individual animals over the study period for farm A (IL-4 and IFN-γ). Fig. S5. PBMC cytokine transcription responses to mitogen/antigen stimulation *in vitro* for individual animals over the study period for farm A (IL-2 and IL-5). Fig. S6. PBMC regulatory cytokine production to mitogen/antigen stimulation *in vitro* for individual animals over the study period for farm A (IL-10 and TGF-β). Fig. S7. Residual plots and qq-normality plots for multivariable linear mixed-effect models with antibody PP value as the response variable. Fig. S8. Residual plots and qq-normality plots for multivariable linear mixed-effect models with change in antibody PP value as the response variable.

  PDF, 939K
