# Supplementary material for: Dairy Heifers Naturally Exposed to Fasciola hepatica Develop a Type 2 Immune Response and Concomitant Suppression of Leukocyte Proliferation
Source: Infect Immun. 2017 Dec 19;86(1):e00607-17. doi: 10.1128/IAI.00607-17 (PMC5736823; doi:10.1128/IAI.00607-17)
Supplement: Supplemental material [file IAI.00607-17_zii012172238s1.pdf]

Supplementary material(s)

The following plots summarise the complete immunological dataset used in the multivariable linear mixed-effects models described in the main body of the paper (Table 3). All plots are shown as time series for individual animals by month of sampling.

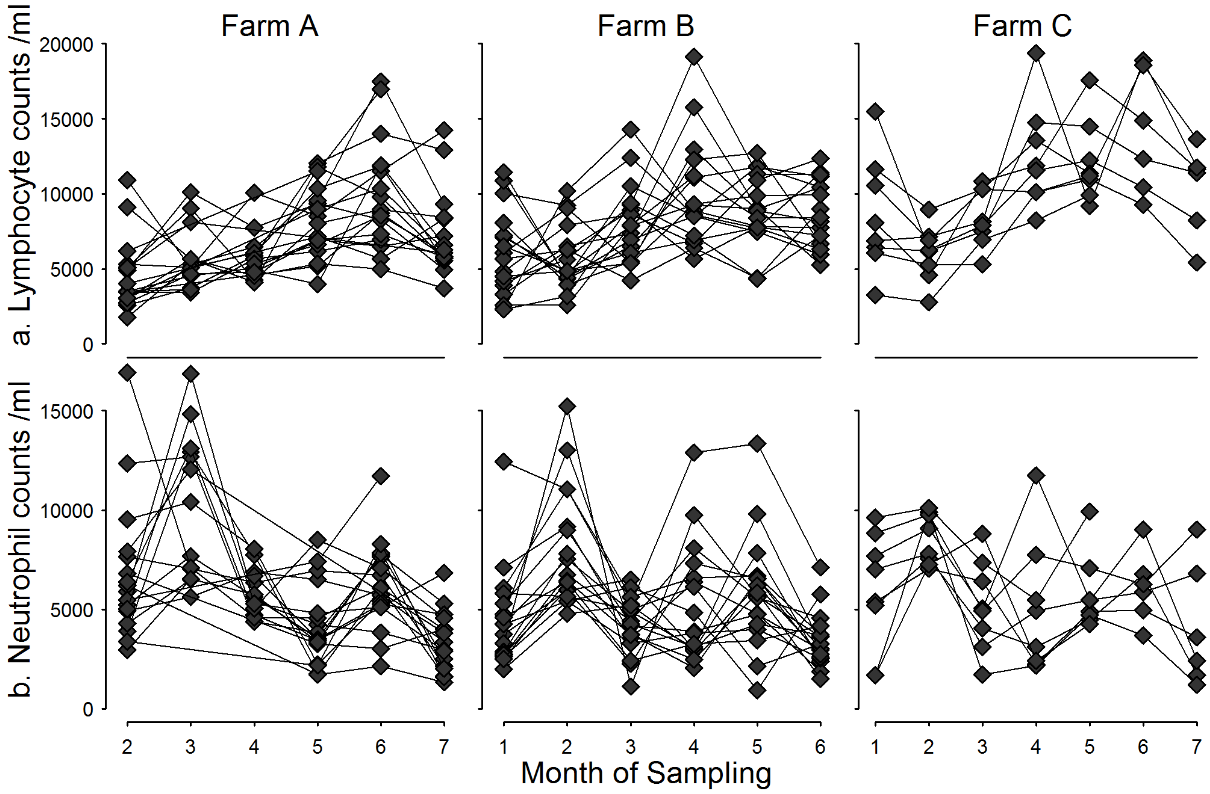

Figure S1: Differential cell counts of individual animals over the study period for each farm (A, B & C). (a.) Lymphoid cells and (b.) Neutrophil counts in peripheral blood. Month of sampling denotes the time point for each sequential sampling visit on each farm.

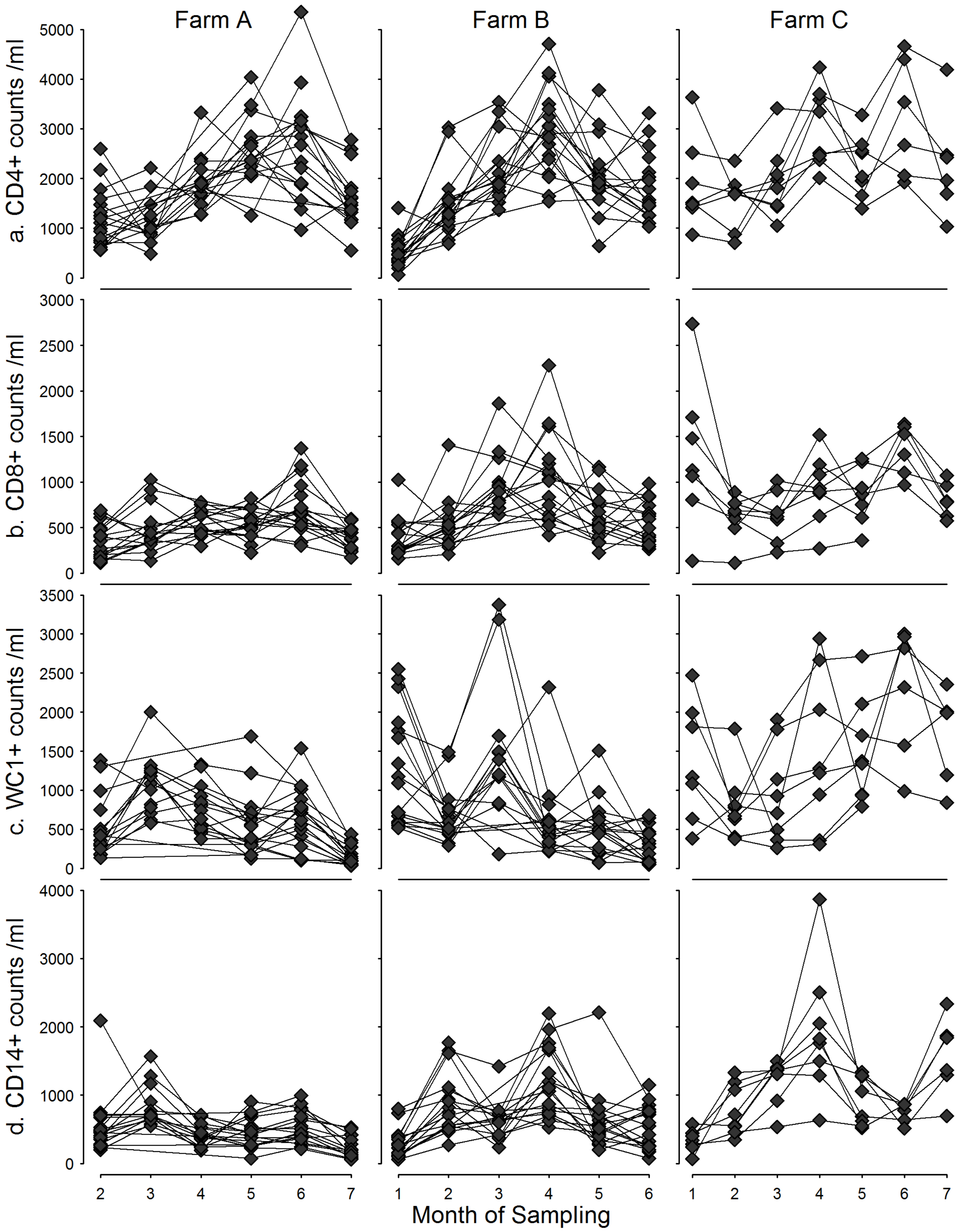

Figure S2: Differential PBMC phenotype counts of individual animals over the study period for each farm (A, B & C). (a.) CD4^{+} cell, (b.) CD8^{+} cell, (c.) WC1^{+} cell, and (d.) CD14^{+} cell counts in PBMCs isolated from peripheral blood. Month of sampling denotes the time point for each sequential sampling visit on each farm.

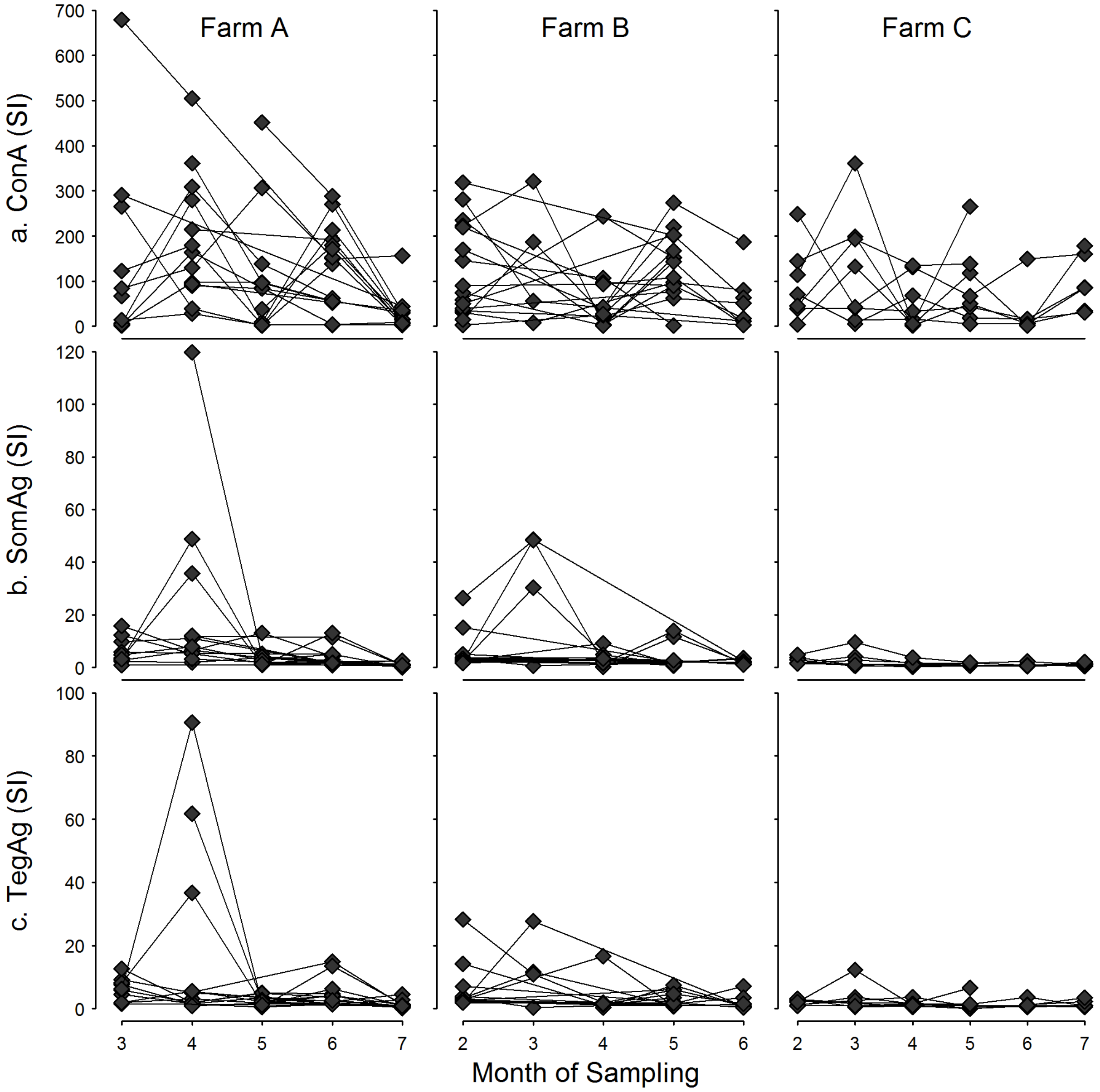

Figure S3: PBMC proliferative responses to mitogen/antigen stimulation *in vitro* for individual animals over the study period for each farm (A, B & C). (a.) Concanavalin A (ConA)-, (b.) *F. hepatica* somatic antigen (SomAg)- & (c.) *F. hepatica* tegumental antigen (TegAg)-stimulated proliferation responses by PBMCs isolated from peripheral blood *in vitro*. Month of sampling denotes the time point for each sequential sampling visit on each farm. PBMC proliferation is expressed as stimulation index (SI), a fold increase increase in [H^3] tritiated thymidine uptake relative to medium controls.

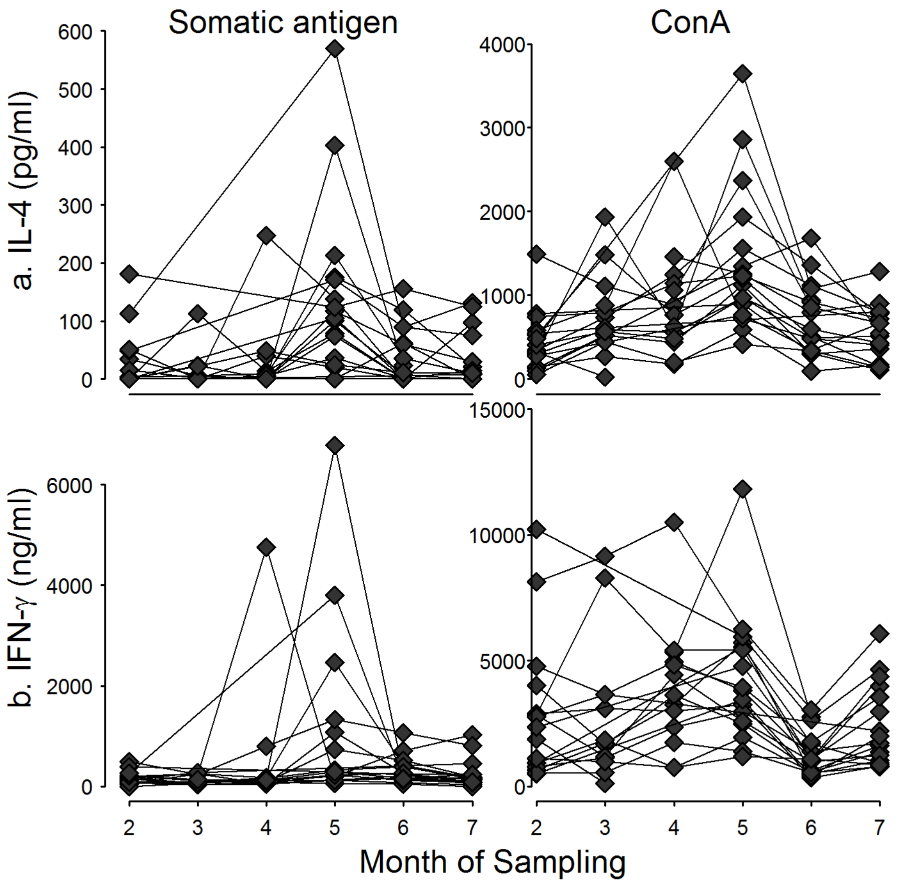

Figure S4: PBMC cytokine production responses to mitogen/antigen stimulation *in vitro* for individual animals over the study period for farm A. (a.) Interleukin(IL)-4 and (b.) Interferon(IFN)-γ production by PBMCs isolated from peripheral blood *in vitro*. Month of sampling denotes the time point for each sequential sampling visit on each farm.

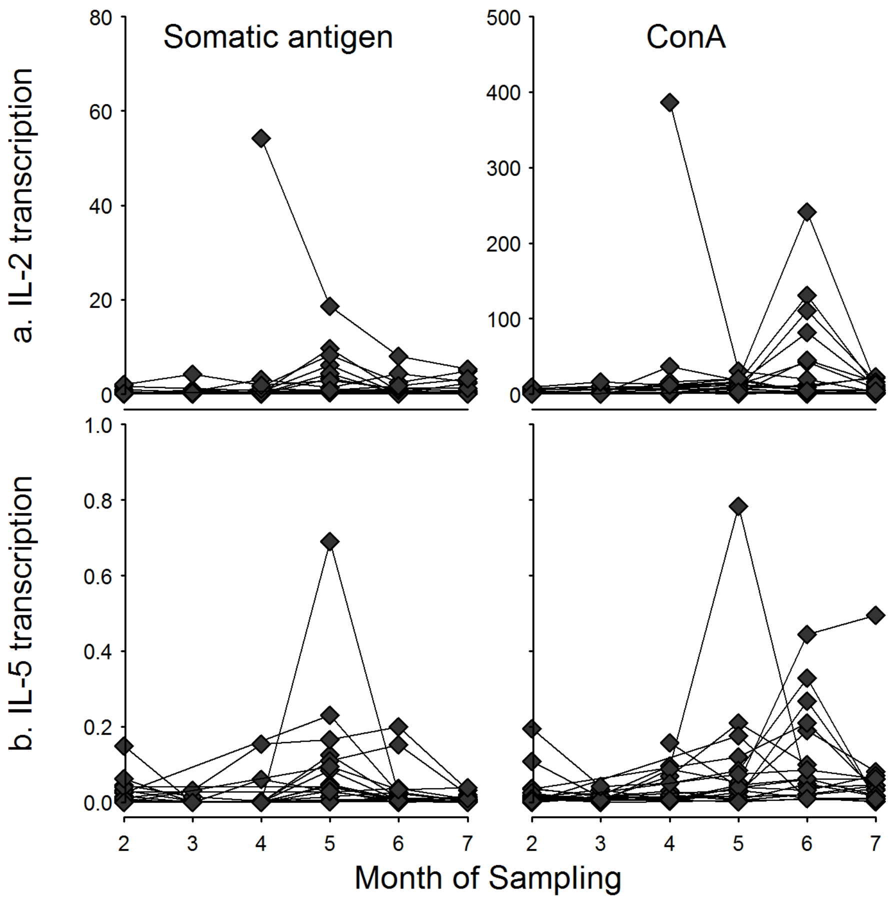

Figure S5: PBMC cytokine transcription responses to mitogen/antigen stimulation *in vitro* for individual animals over the study period for farm A. (a.) Interleukin(IL)-2 and (b.) Interleukin(IL)-5 transcription by PBMCs isolated from peripheral blood *in vitro*. Month of sampling denotes the time point for each sequential sampling visit on each farm. Cytokine mRNA transcription is given as relative transcription against the expression of ribosomal 28s housekeeper gene.

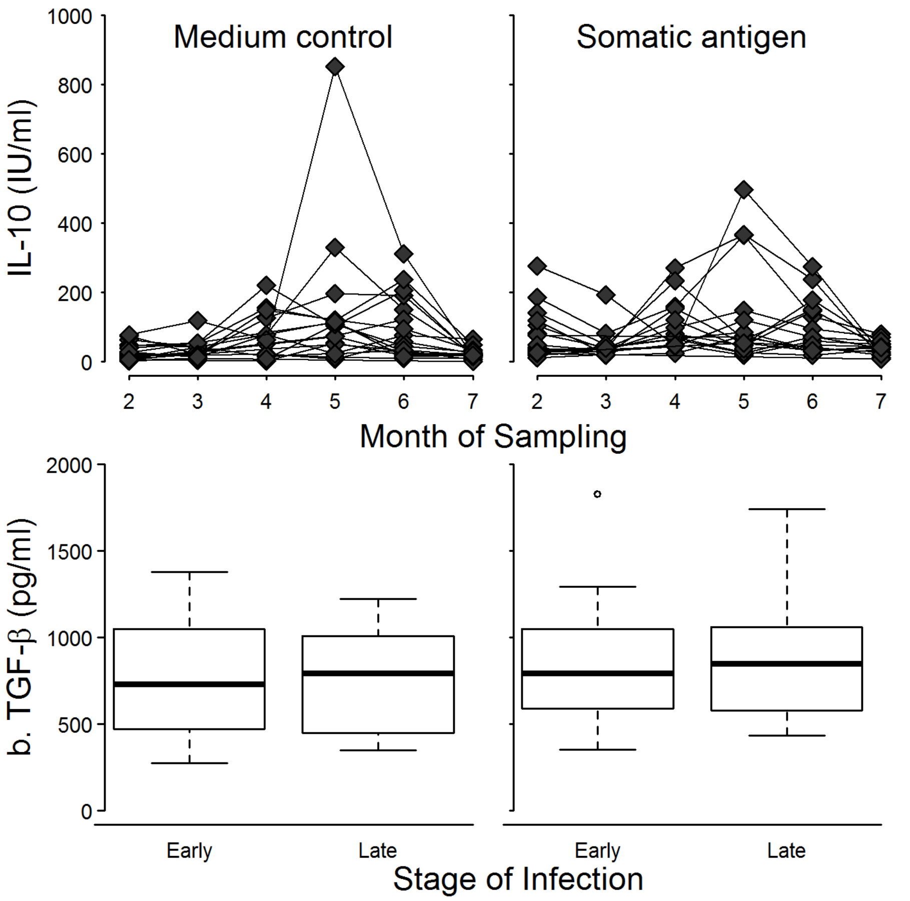

Figure S6: PBMC regulatory cytokine production to mitogen/antigen stimulation *in vitro* for individual animals over the study period for farm A. (a.) Interleukin(IL)-10 and (b.) Transforming Growth Factor(TGF)-β production by PBMCs isolated from peripheral blood *in vitro*. For IL-10, month of sampling denotes the time point for each sequential sampling visit on each farm. For TGF-β, early and late stages of infection refers to the timing of infection for each paired sample analysed; samples for each individual animal were selected at, or close to sero-conversion to represent early stage infection, and from the last available time point to represent chronic stages of infection.

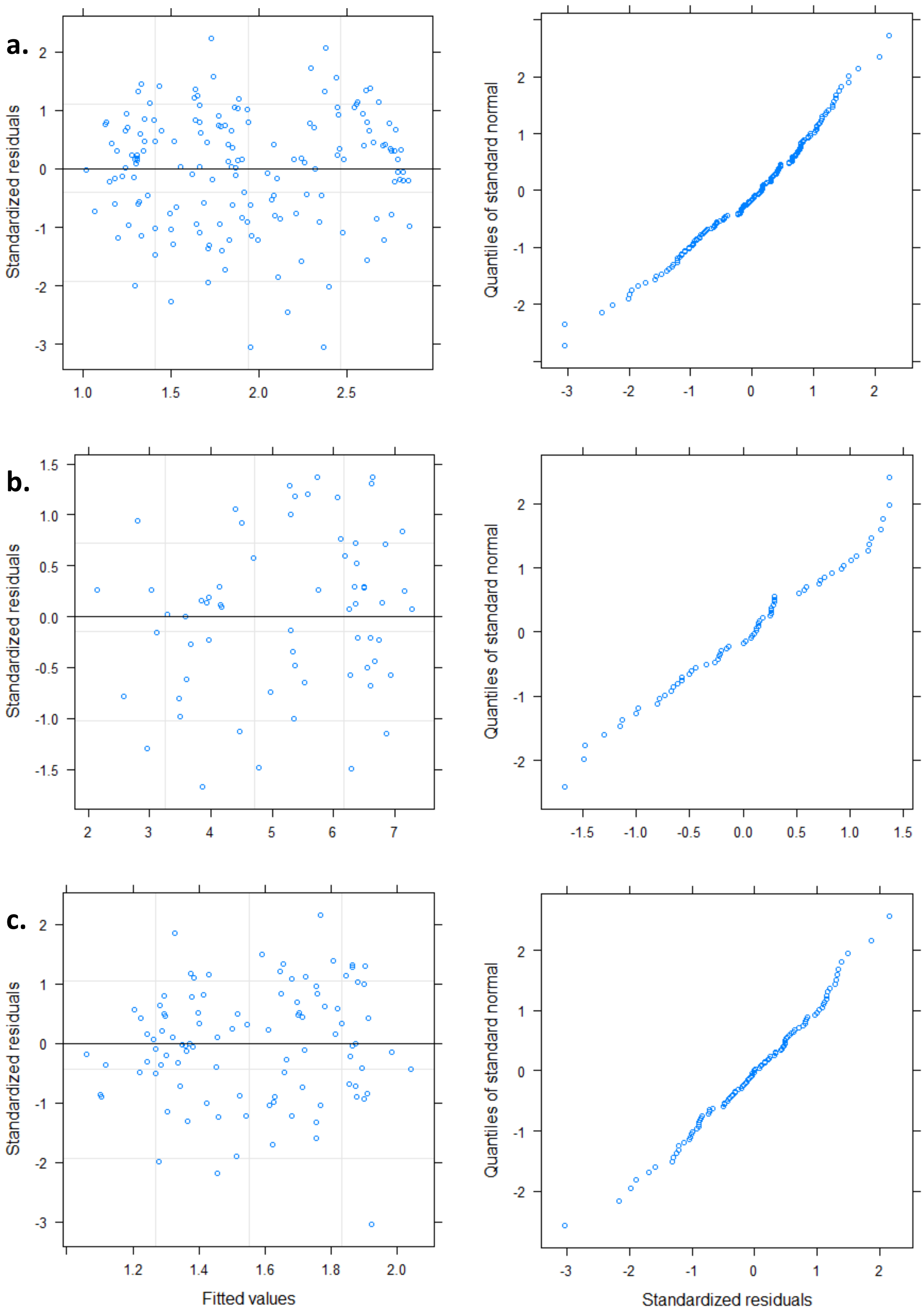

Figure S7: Residual plots (left) and qq-normality plots (right) for multivariable linear mixed-effect models with antibody PP-value (Ab) as the response variable (Y). a.) lme_1, b.) lme_2 and c.) lme_3 as described in the main body of the paper (Table 1).

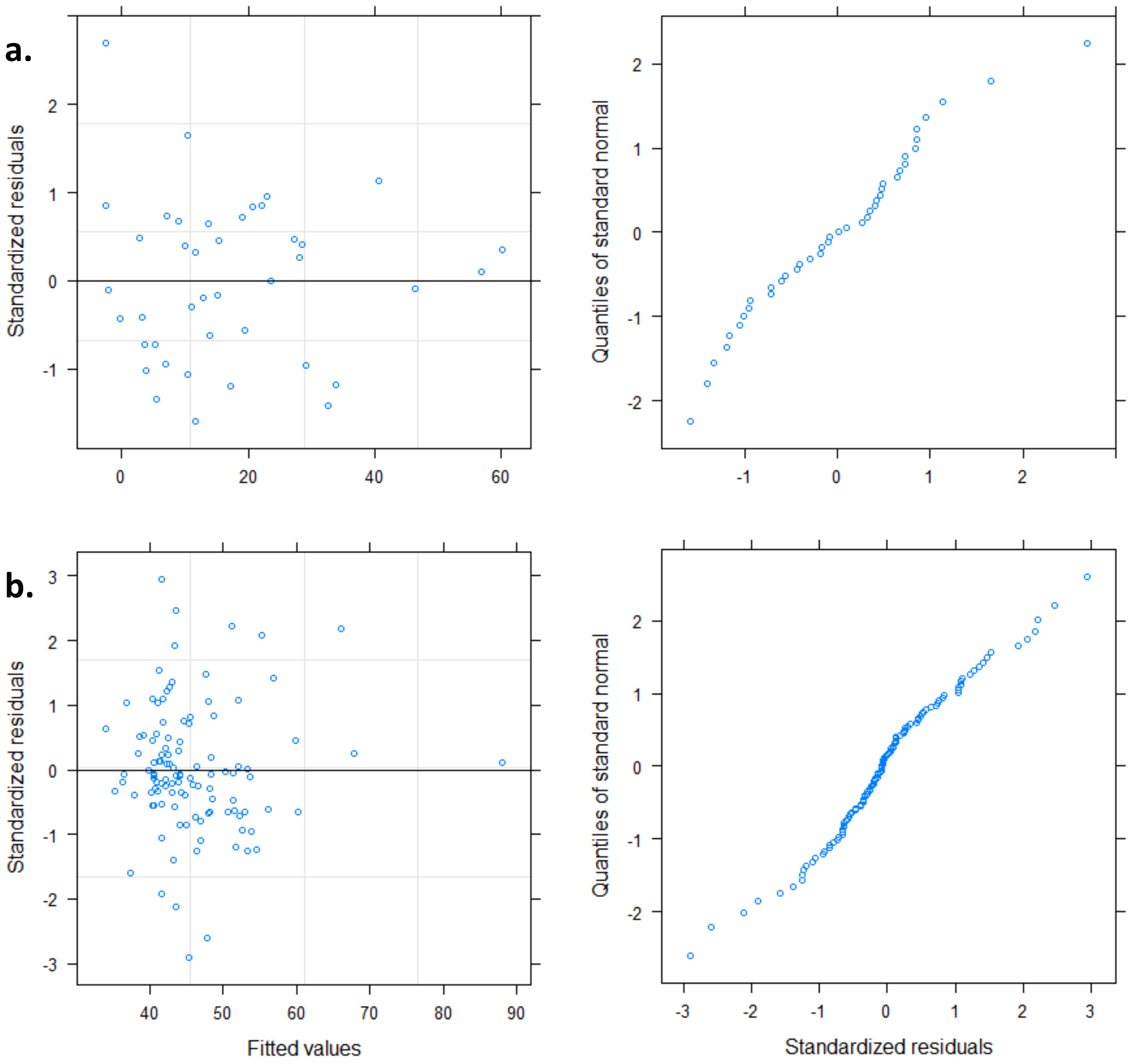

Figure S8: Residual plots (left) and qq-normality plots (right) for multivariable linear mixed-effect models with change in antibody PP-value (ΔAb) as the response variable (Y). a.) lme_4 and b.) lme_5 as described in the main body of the paper (Table 1).
